# Computer-based automation of concentration measurements with ion-selective electrodes

**DOI:** 10.1155/S1463924689000283

**Published:** 1989

**Authors:** josé A. Ibañez, L. Victoria, Rafael M. Barzanallana

**Affiliations:** 1 Dept. Física Aplicada Facultad de Ciencias Químicas y Matemáticas Universidad de Murcia Murcia 30071 Spain; 2 Dept. Informática y Automática Universidad de Murcia Murcia 30071 Spain

## Abstract

An integrated computer system consisting of an expandable ionanalyzer and a PC has been used to automate concentration measurements with ion-selective electrodes (ISEs). The ionanalyzer determines ionic concentrations using a reference electrode coupled with an ISE. The measurements and calibrations are carried out in a thermostated sample changer equipped with a flow cell. The data obtained by the ionanalyzer are sent via a standard RS 232-C interface to a microcomputer. In this paper, we describe the automatic data acquisition system and the subsequent processing of the measurements. One program (Transorion) automatically collects the measurements carried out by the ionanalyzer, giving a real-time graphic representation of the measurement on the computer screen. A second program (Graforion) facilitates the management of the data stored by the first program, and listing and graphics of these can be obtained on the printer/plotter. The method has been used to study continuous concentration changes in an aqueous solution of potassium iodide.


Journal of Automatic Chemistry Vol. 11, No. 3 (May–June 1989), pp. 129–133

### Computer-based automation of concentration measurements with ion-selective electrodes

**José A. Ibañez,† L. Victoria† and Rafael M. Barzanallana‡**

† *Dept. Física Aplicada, Facultad de Ciencias Químicas y Matemáticas, Universidad de Murcia, 30071 Murcia, Spain*

‡ *Dept. Informática y Automática, Universidad de Murcia, 30071 Murcia, Spain*

*An integrated computer system consisting of an expandable ionanalyzer and a PC has been used to automate concentration measurements with ion-selective electrodes (ISEs). The ionanalyzer determines ionic concentrations using a reference electrode coupled with an ISE. The measurements and calibrations are carried out in a thermostated sample changer equipped with a flow cell. The data obtained by the ionanalyzer are sent via a standard RS 232-C interface to a microcomputer. In this paper, we describe the automatic data acquisition system and the subsequent processing of the measurements. One program (Transorion) automatically collects the measurements carried out by the ionanalyzer, giving a real-time graphic representation of the measurement on the computer screen. A second program (Graforion) facilitates the management of the data stored by the first program, and listing and graphics of these can be obtained on the printer/plotter. The method has been used to study continuous concentration changes in an aqueous solution of potassium iodide.*

### Introduction

This paper reports an automatic method for the measurement of ionic concentrations using ion-selective electrodes (ISEs). Ionic concentrations are measured directly by an expandable ionanalyzer with an RS 232C (CCITT V24) output, which gives the concentration, the corresponding electrical potential difference and facilitates the necessary ISE calibrations. The measurements carried out by the ionanalyzer are received by a microcomputer using a program (Transorion), and can be stored on a hard or floppy disk and displayed on the computer screen. A second program (Graforion) gives the data read-out, listing the values obtained and/or providing graphics.

### Experimental

The experimental device consists of a reactor (with stirring system), a liquid circulation system, a thermostat circuit, a measurement system (consisting of electrodes, a sample changer and the ionanalyzer and a data collection and recording system.

*Reactor*

The solution to be analyzed was placed in a thermostated reactor. The solution was homogenized using a paddle-stirrer driven by a variable-speed motor, which was controlled by an electro-optical device; we used a stirring rate of 100 rev $min^{-1}$. The experiment was conducted at 25·0 ± 0·1 °C, with an aqueous solution of potassium iodide at an initial concentration of 0·01 M; this concentration was increased by adding a 0·1 M solution of the same electrolyte from a reservoir (M1 in figure 1), keeping the volume of the solution in the reactor (R) constant by extraction to a waste reservoir (M2). A peristaltic pump

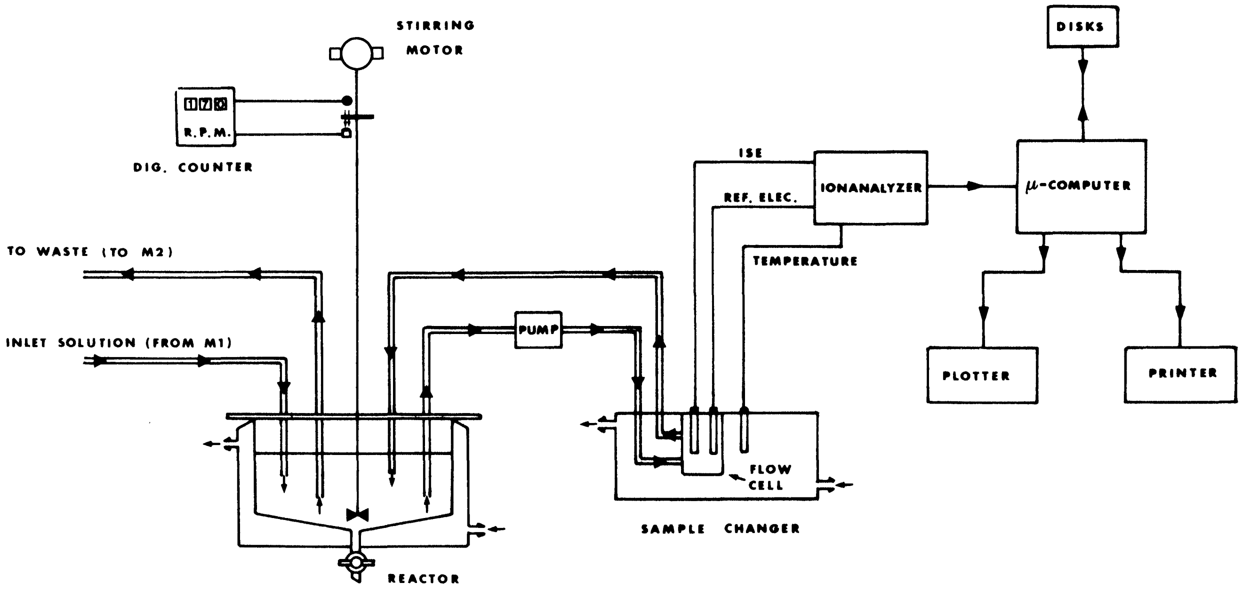

*Figure 1. Schematic diagram of the apparatus.*

0142–0453/89 $3.00 © 1989 Taylor & Francis Ltd.

and a compensating system to eliminate any possible difference between inlet and outlet volumes were required. In this way the concentration of the electrolyte was increased. After reaching a maximum value, the solution was returned to the original 0·01 M concentration by continuous dilution.

*Liquid circulation system*

This circuit consisted of a four-channel peristaltic pump (Gilson-Minipuls), in which only two channels were used (one from M1 and the other to M2). The transport tubes were previously calibrated to obtain equal flows in the two channels. An electro-optical level controller was necessary to maintain equilibrium between the inlet and outlet flows [1].

*Thermostat circuit*

This was a double circuit used for both heating and cooling; these alternative functions could be selected by manually turning valves. The heating circuit has a thermostated bath with a temperature display and a pump. In the cooling circuit, an appropriate device reduces the temperature of the coolant inside a container with insulating walls, from where it flows through the circuit.

*Measurement system*

To measure concentration changes in aqueous potassium iodide solutions, an ISE for $I^-$ (Orion, Model 94-53BN) was used with a double-union reference electrode (Orion, Model 90-02). The ISE has a linear range from 1 to 10·5 M. As electrodes require periodical calibration we have designed a sample changer thermostated in parallel with the reactor, with six magnetic stirring vessels for standard solutions, one of which also acts as a wash vessel, and a flow cell in which the electrodes are placed (figure 2). This electrode response is fed to an ionanalyzer (Orion, EA940) where the temperature, the concentration and the associated electrical potential difference are displayed in a continuous form. By means of an output RS 232C the data obtained are sent to a microcomputer, where they are stored for further use (figure 1).

*Data acquisition and recording system*

An information system is used for acquiring, storing and processing the data. This system consists of a microcomputer (Olivetti M24) with three RS 232C interfaces, a high resolution graphics screen, a printer (Olivetti DM 286/2) and a plotter (Roland DXY 880); the operating system is MS DOS. A program (Transorion) automatically collects the measurements carried out by the ionanalyzer. It manages the serial asynchronous communications interface and continually receives and processes the data received from the ionanalyzer. When these data make up a complete measurement the program stores and displays them graphically on the screen. The transmission of this information can be interrupted at any time so that additional information can be entered through the keyboard or electrode calibrations carried out. It is also possible to interrupt the program to return to the main menu. Hence the program allows the real time graphic representation of the measurements and can be interrupted at any moment to realize other functions.

Figure 3 shows the flowchart of the Transorion and the complete development of the program. This program, written in GW Basic language, is composed of the following parts: Definition of constants and input of necessary parameters through the keyboard; communica-

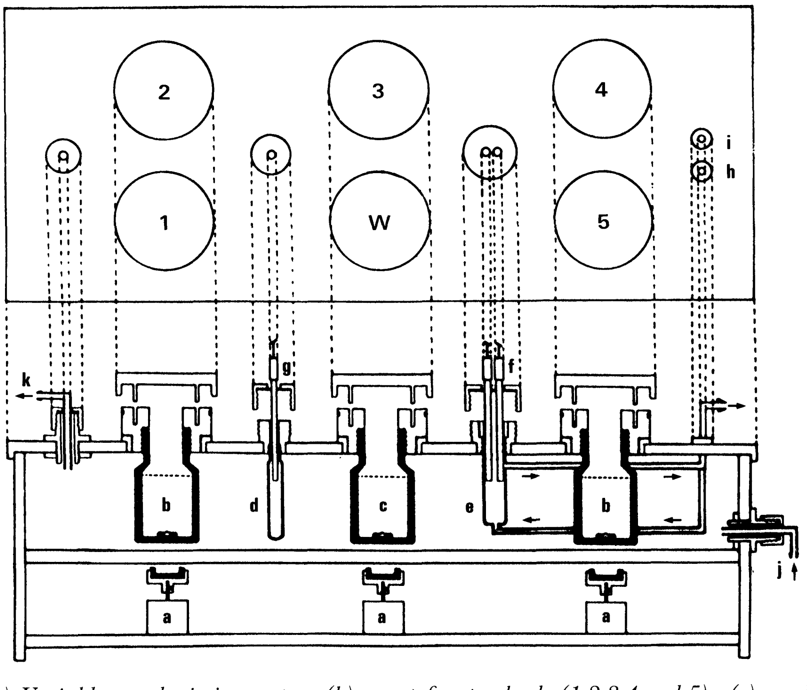

*Figure 2. Sample changer. (a) Variable speed stirring motor; (b) vessel for standards (1,2,3,4 and 5); (c) wash vessel (W); (d) cell for temperature probe; (e) measurement flow cell; (f) electrodes (reference and ISE); (g) temperature control probe; (h) sample outlet; (i) sample inlet; (j) inlet for thermostating liquid; and (k) outlet for thermostating liquid.*

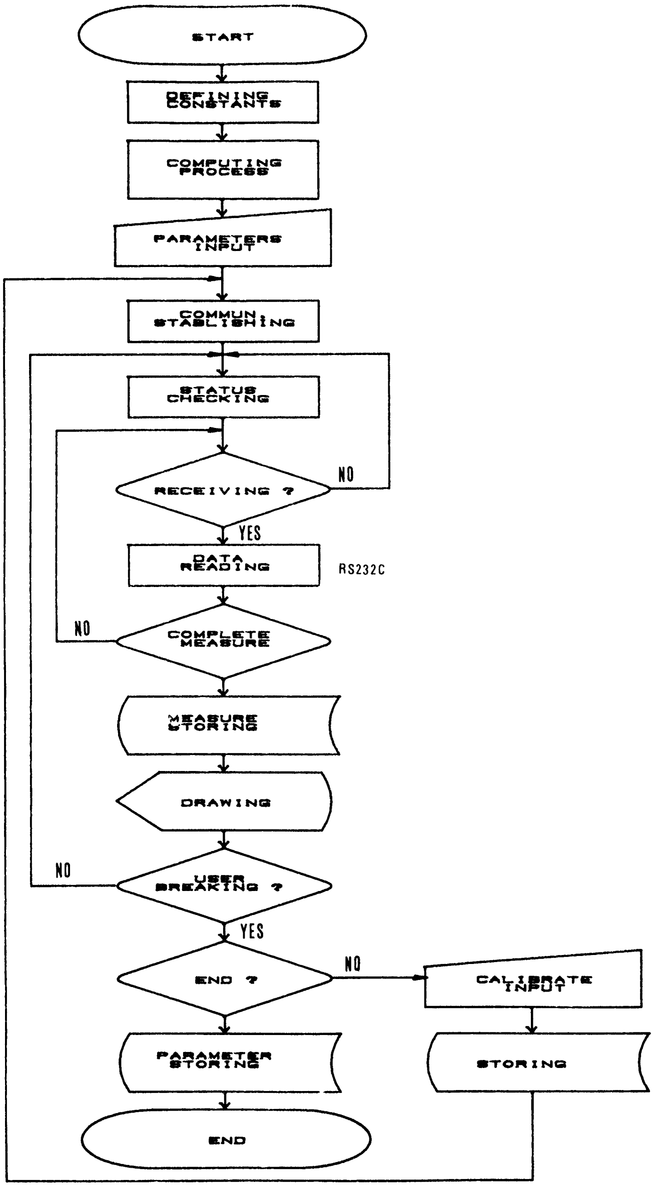

"TRANSORION"

*Figure 3. Flowchart of Transorion program for data acquisition.*

tions status checking through RS 232C interface (when communication is not well established, the screen indicates that there is a fault, generally due to connection cables); data input through the interface and data processing and representation on screen; and storage on disk of the values and the time of each measurement.

The user can interrupt the measurement in order to carry out any other process (e.g. electrode calibration). When calibrating the electrodes, it is possible to store on disk the calibration information, and when the user considers the process to be finished, he can return to the main menu.

A second program (Graforion) facilitates the management of the data stored by the Transorion program and its task is to list and display graphically the measurements obtained. When a file name is given by the user, this second program enables him to extract the file and

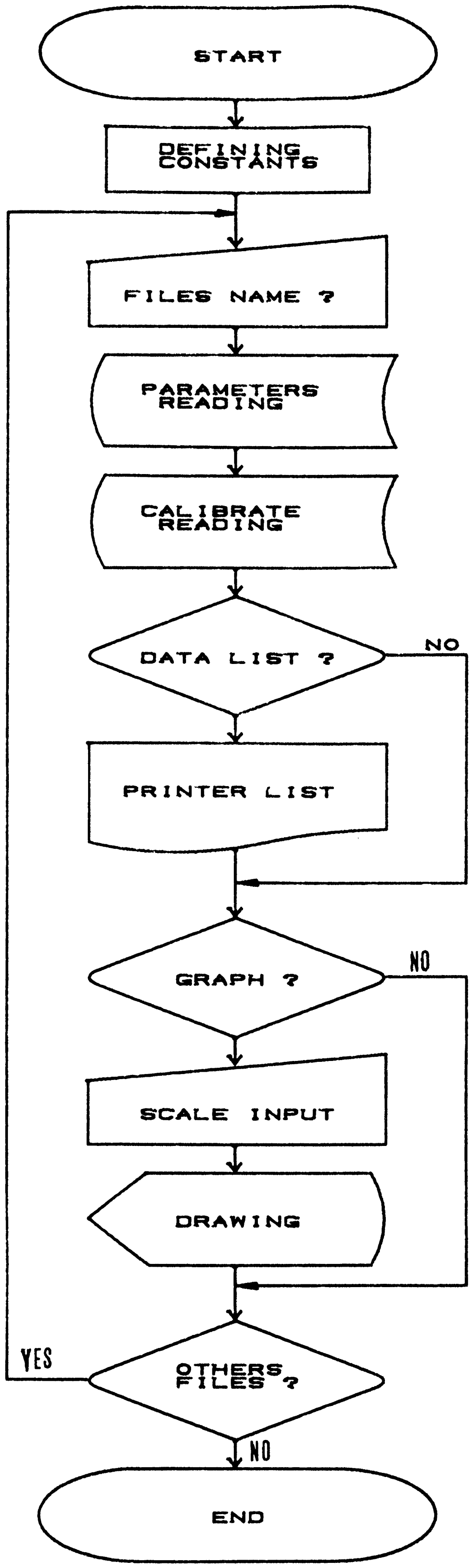

"GRAFORION"

*Figure 4. Flowchart of Graforion program for data processing.*

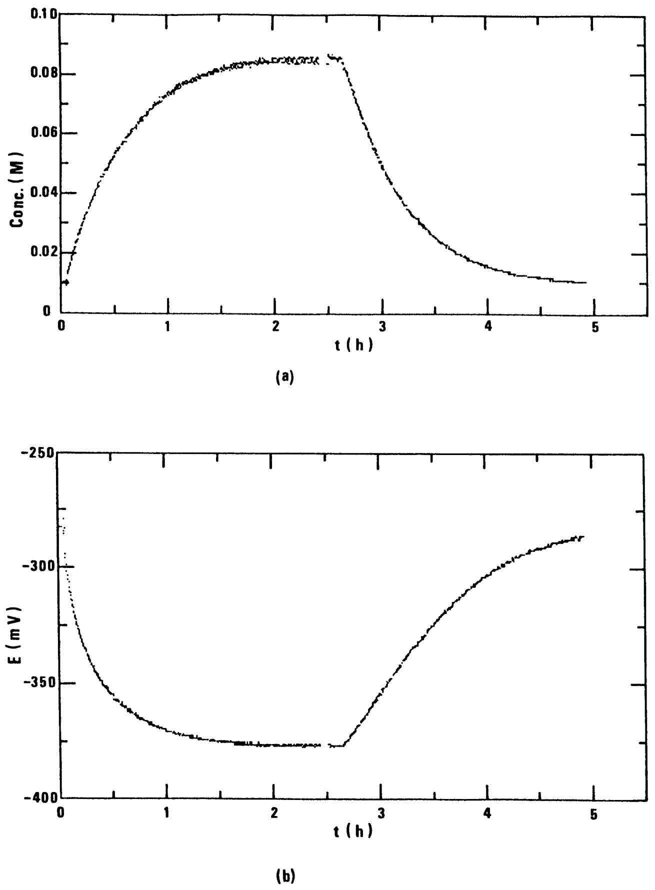

*Figure 5. (a) Concentration of the sample as a function of time. (b) Electrical potential difference between ISE and reference electrode for the problem solution versus time. Graphics produced by printer.*

see the characteristic parameters of the process and the values of measurements at specific times, generating a total or partial listing and a graphic representation of the measurements. Alternatively, a copy can be obtained by printer or plotter. Figure 4 shows the flowchart of the Graforion program. When the process is over it is possible to return to the beginning of the program to select another file without returning to the main menu.

### Results

The method developed has been applied to the study of the concentration change in an aqueous solution of potassium iodide prepared with doubly distilled, degassed and deionized water, with an initial value of 0·01 M. The solution was continuously renewed by a peristaltic pump, which extracted a volume of solution with a tube while introducing by another tube the same volume of solution at 0·1 M; the reverse process (from 0·1 to 0·01 M) was then carried out.

The concentration measures were continuously obtained with an ISE versus a reference electrode over a period of approximately 5 h. Concentration changes with time for the solution inside the reactor were recorded. The results obtained are shown in figure 5.

Table 1 shows part of the listing obtained by the printer. This part corresponds to the start of the experiment and, as can be seen, specifies the number and the time corresponding to the measurement, the concentration at

*Table 1. Partial data listing corresponding to electrical potential difference and concentration of the sample. Calibration range: 0·005–0·1 M. Slope: −58·5 mV/decade. Ordinate intercept: −410·5 mV.*

| Measurement number | Potential/ mV | Concentration/ M | Time/ s | Temperature, °C |
|---|---|---|---|---|
| 1 | −292·90 | 0·9780E-02 | 13 | 25·50 |
| 2 | −292·90 | 0·9820E-02 | 33 | 25·50 |
| 3 | −292·90 | 0·9780E-02 | 48 | 25·50 |
| 4 | −292·90 | 0·9780E-02 | 66 | 25·50 |
| 5 | −293·00 | 0·9820E-02 | 81 | 25·50 |
| 6 | −293·00 | 0·9820E-02 | 96 | 25·50 |
| 7 | −293·20 | 0·9900E-02 | 111 | 25·50 |
| 8 | −292·90 | 0·9780E-02 | 128 | 25·50 |
| 9 | −293·00 | 0·9820E-02 | 143 | 25·50 |
| 10 | −293·20 | 0·9900E-02 | 160 | 25·50 |
| 11 | −293·00 | 0·9820E-02 | 175 | 25·50 |
| 12 | −293·70 | 0·1010E-01 | 192 | 25·40 |
| 13 | −294·60 | 0·1050E-01 | 209 | 25·40 |
| 14 | −290·90 | 0·9040E-02 | 225 | 25·50 |
| 15 | −293·50 | 0·1000E-01 | 242 | 25·50 |
| 16 | −299·70 | 0·1280E-01 | 257 | 25·50 |
| 17 | −300·50 | 0·1320E-01 | 273 | 25·50 |
| 18 | −302·00 | 0·1400E-01 | 289 | 25·50 |
| 19 | −302·90 | 0·1450E-01 | 304 | 25·40 |
| 20 | −303·80 | 0·1500E-01 | 320 | 25·50 |
| 21 | −304·80 | 0·1560E-01 | 336 | 25·40 |
| 22 | −305·70 | 0·1620E-01 | 351 | 25·50 |
| 23 | −305·60 | 0·1610E-01 | 368 | 25·50 |
| 24 | −306·60 | 0·1680E-01 | 384 | 25·50 |
| 25 | −307·80 | 0·1760E-01 | 401 | 25·40 |

this time, the associated electrical potential difference and, finally, the temperature.

### Conclusion

The technique described in this paper is an efficient and reliable method for measuring concentration changes with ISEs. The incorporation of a sample changer with measuring cell, thermostated in parallel with the reactor, allows the periodic calibration of ISEs and the measurement of problem concentrations at the same temperature, and can therefore eliminate errors.

### Acknowledgement

The authors acknowledge financial support from the CICYT of the Spanish government under grant No. PB85-0240-C02-02.

### References

1. Ibáñez, J. A. Victoria, L., Ortega-Navas, J. Hernández, A. and Tejerina, F., *Anales de Física (B)*, **82** (1986), 213.

### Calendar

### JUNE

25–30 June 1989 *Eurosensors III and 5th International Conference on Sensors and Actuators: Montreux, Switzerland.* Contact Eurosensors (Transducers '89), COMST S.A., PO Box 415, 1001 Lausanne 1, Switzerland.

### JULY

30 July–5 August 1989 *SAC 89: Cambridge, UK.* Contact Analytical Division, Royal Society of Chemistry, Burlington House, Piccadilly, London W1V 0BN.

### SEPTEMBER

11–13 September 1989 *RSC/SCI/IOP Surface Analysis Techniques and Applications: Manchester, UK.* Contact Mrs E. S. Wellingham, Field End House, Bude Close, Nailsea, Bristol BS19 2FQ.

20–22 September 1989 *International Symposium on Detection in Liquid Chromatography and Flow Injection Analysis: Córdoba, Spain.* Contact Dr M. D. Luque de Castro, Dpto. Quimica Analitica, Facultad de Ciencias, 14004 Córdoba, Spain.

25–27 September 1989 *Sensors and their Applications IV: Canterbury, UK.* The Meetings Officer, Institute of Physics, 47 Belgrave Square, London SW1X 8QX.

25–28 September 1989 *Third International Symposium on Kinetics in Analytical Chemistry: Dubrovnik, Yugoslavia.* Contact Professor Gordana A. Milonović. Department of Chemistry, University of Belgrade, KAC PO Box 550, 11001 Belgrade, Yugoslavia.

26–28 September 1989 *The British Laboratory Week: Olympia, London.* Including Laboratory 89, Computer Aided Sciences, Bio 89, Medical Laboratory Sciences and Analyticon. Contact Curtis Steadman and Partners, The Hub, Emson Close, Saffron Walden, Essex CB10 1HL.

### 1990
### MARCH

5–9 March 1990 *41st Pittsburgh Conference and Exposition on Analytical Chemistry and Applied Spectroscopy: New York, USA.* Contact The Pittsburgh Conference, 300 Penn Center Boulevard, Suite 332, Pittsburgh PA 15235, USA.

### AUGUST

26–31 August 1990 *Euroanalysis VII: Vienna, Austria.* Contact Prof. Dr M. Grasserbauer, c/o Interconvention, Austria Center Vienna, A-1450 Vienna, Austria.

